# Treatment of Inpatient Opioid Withdrawal with Short-Acting Full Agonist Opioids at a Safety-Net Hospital

**DOI:** 10.1007/s11606-024-09321-5

**Published:** 2025-01-06

**Authors:** Gabriela Steiner, Leslie W. Suen, Marlene Martin, Sasha Skinner, Pierre Crouch, Oanh K. Nguyen, Kristin Slown, Alexander Logan

**Affiliations:** 1 University of California, San Francisco, 1001 Potrero Avenue, Bldg 5 Rm 5H06, San Francisco, CA 94110 USA; 2 Zuckerberg San Francisco General Hospital and Trauma Center, San Francisco, USA

**Keywords:** substance use disorder, opioid use disorder, medications for opioid use disorder, ppioid withdrawal syndrome

## Abstract

**Background:**

Fentanyl use leads to increased opioid tolerance in people with opioid use disorder, complicating management of opioid withdrawal syndrome. While accepted as gold standard, methadone and buprenorphine may be insufficient to treat acute opioid withdrawal. Short-acting full agonist opioids (SAFAO) may improve treatment in the acute care setting.

**Aim:**

Characterize use of SAFAO to treat opioid withdrawal syndrome for inpatients.

**Setting:**

Urban safety-net hospital.

**Participants:**

Inpatients with opioid use disorder.

**Program Description:**

Addiction Consult Team offers SAFAO to manage opioid withdrawal syndrome.

**Program Evaluation:**

We performed retrospective chart review of hospitalizations (*n* = 124) for patients with opioid use disorder who received SAFAO between March and June 2023. Patients received methadone or buprenorphine during 94% of hospitalizations. On average, 278 mg (SD 407 mg) oral morphine equivalents (OMEs) of SAFAO were administered daily during the first hospitalization week. Daily Clinical Opiate Withdrawal Scale scores and SAFAO OMEs were inversely correlated (Spearman rank correlation = − 0.96, *p* = 0.003). Five hospitalizations exhibited adverse events (AEs).

**Discussion:**

Use of methadone/buprenorphine did not alleviate the need for SAFAO, suggesting that SAFAO administration may be an important intervention for opioid withdrawal. Use of escalating OMEs of SAFAO was associated with reduced withdrawal severity. This practice was well-tolerated with few AEs.

**Supplementary Information:**

The online version contains supplementary material available at 10.1007/s11606-024-09321-5.

## INTRODUCTION

Potent synthetic opioids such as fentanyl have increasingly dominated the illicit drug market in North America, leading to high opioid tolerance in patients with opioid use disorder.^1^ Consequently, these patients may experience more severe opioid withdrawal syndrome while hospitalized. Patients with opioid use disorder are nearly three times more likely to have patient-directed discharge than patients without opioid use disorder, and qualitative studies have shown that inadequate treatment of opioid withdrawal syndrome contributes to this disparity.^2–5^ These premature discharges contribute to higher all-cause mortality and readmission rates, underscoring the need for interventions that adequately treat opioid withdrawal syndrome in the inpatient setting.^6–8^

The American Society of Addiction Medicine guidelines recommend treating acute opioid withdrawal syndrome with methadone, buprenorphine, and/or non-opioid adjuncts.^9^ Medications for opioid use disorder, including methadone and buprenorphine, are gold standard treatments but can be insufficient to treat acute opioid withdrawal syndrome, particularly among patients using fentanyl.^10^ Consequently, using short-acting full agonist opioids (SAFAO) to treat opioid withdrawal syndrome during hospitalization has recently gained interest, as these medications can provide timely relief in the first few days of hospitalization when withdrawal peaks.^11–14^ Since 2019, our Addiction Consult Team (ACT) has offered SAFAO in addition to medications for opioid use disorder and non-opioid adjuncts to treat opioid withdrawal syndrome in hospitalized patients who endorse daily heroin or fentanyl use.

Use of SAFAO to treat opioid withdrawal syndrome may allow for more effective, patient-centered care. However, given the relative novelty of this practice, limited data are available to support widespread implementation. Additionally, there is disagreement in the literature regarding the role of SAFAO for the management of opioid withdrawal syndrome, highlighting the need for more robust evidence.^10^

In this study, we characterize how SAFAO were used to treat inpatient opioid withdrawal syndrome at an urban, safety-net hospital. We describe (1) the population and hospitalization characteristics of patients receiving SAFAO, (2) SAFAO doses used to manage opioid withdrawal syndrome, and (3) the number and nature of adverse events among patients who received SAFAO.

## SETTING AND PARTICIPANTS

This effort to treat opioid withdrawal syndrome using SAFAO was enacted by the ACT at an urban safety-net hospital where one-third of patients have a substance use disorder.^15^ ACT is an interprofessional service that provides expert consultation focused on substance use disorder diagnosis, medication for addiction treatment, harm reduction, and referral for ongoing treatment.^16^

## PROGRAM DESCRIPTION

Since 2019, ACT has initiated SAFAO for inpatients with opioid withdrawal syndrome, in addition to medications for opioid use disorder and non-opioid adjuncts. In 2023, ACT developed internal guidelines (Supplement 1) to reduce practice variation and educated front-line clinicians regarding rationale, patient selection, and best practices for treating opioid withdrawal syndrome with SAFAO through nursing huddles, resident noon conferences, and department meetings.

ACT identifies SAFAO candidates following referral by the primary team and/or a positive screen for unhealthy substance use, conducted upon admission for all patients.^17^ ACT offers SAFAO to all patients with opioid withdrawal syndrome who endorse daily fentanyl and/or heroin use, regardless of whether they initiate medications for opioid use disorder. When desired, methadone is initiated with a standard titration (maximum dose of 40 mg on day 1, 50 mg on day 2, and 60 mg on days 3–5), while buprenorphine can be initiated with high-dose or low-dose overlap approaches.^17^ Clinicians titrate SAFAO throughout hospitalization to relieve opioid withdrawal syndrome while avoiding oversedation, guided by physical exam, Clinical Opiate Withdrawal Scale (COWS) scores, and subjective symptoms.

## PROGRAM EVALUATION

We conducted a structured retrospective chart review of electronic health records (EHR) for adult inpatients with opioid use disorder who received SAFAO between March and June 2023. We included hospitalizations with International Classification of Diseases 10 (ICD-10) F11 codes for opioid use disorder who were seen by ACT, received SAFAO during the first week of hospitalization per pharmacy data, and endorsed daily fentanyl or heroin use as documented in their medical record. We excluded hospitalizations less than 24 h and those involving patients in custody. The University of California San Francisco Institutional Review Board exempted this study from review.

We extracted patient socio-demographics, substance use disorder histories, and hospitalization characteristics (e.g., primary service, pregnancy status, surgery during hospitalization, toxicology results, daily maximum COWS, presence of adverse events, and discharge type) from chart review. For patients with multiple hospitalizations, we extracted patient characteristics from the index hospitalization. Primary service was categorized as Medical, Surgical, ICU, or Other. We graded withdrawal severity using the National Institute on Drug Abuse’s guidelines based on COWS scores.^18^ We extracted SAFAO agents used, route, and doses received during the first week of hospitalization from the pharmacy database and calculated oral morphine equivalents (OMEs) of SAFAO for each day using a conversion table from Nielsen et al.^19^

The primary AE of interest was grade 3 (“difficult to arouse”), grade 4 (“life-threatening consequences”), and grade 5 (“death”) sedation, defined by the Common Terminology Criteria for Adverse Events.^20^ Adverse events were identified from significant event and discharge notes and notes identified using the search terms “sedation,” “oversedation,” “sedated,” and “over-sedated.” Secondary adverse events included fall, intubation, and naloxone administration and were identified using relevant search terms.

Regarding statistical tests, we compared hospital discharge types (patient-directed vs regular) according to categories of withdrawal severity using Fisher’s exact test. We compared average daily OMEs between groups of interest (e.g., pregnant/postpartum patients) using a Welch two-sample *t*-test. We used Spearman’s rank correlation to evaluate the relationship between average OMEs and average maximum COWS during the first week of hospitalization. An alpha level of 0.05 was used for all statistical tests. All data processing and statistical analyses were done using R programming language, R Studio, and the tidyverse package.^21–23^

## SOCIODEMOGRAPHIC AND SUBSTANCE USE CHARACTERISTICS

Between March and June 2023, 124 hospitalizations representing 108 unique patients were included. Socio-demographics are presented in Table 1. The primary opioid used was fentanyl in 83% and heroin in 17% of patients; most patients (77%) had previously received medications for opioid use disorder; 79% of patients were diagnosed with an additional substance use disorder, most commonly stimulant use disorder (62%) (Table 1). Among patients who only reported heroin use and received toxicology labs, 54% detected fentanyl (*n* = 7/13).

**Table 1:** Patient and Hospitalization Characteristics for Inpatients Receiving SAFAO for Opioid Withdrawal Syndrome

**Patient characteristics **(***n = 108)***	*N* (%) or mean (SD)
**Age**	42 (11)
**Gender**	
Cisgender male	69 (64%)
Cisgender female	34 (31%)
Transgender female or male	5 (5%)
**Race/ethnicity**	
White	67 (62%)
Black	21 (19%)
Latinx	15 (14%)
Other	5 (5%)
**Unhoused**	81 (75%)
**Primary insurance**	
Medicaid	95 (88%)
Medicare	10 (9%)
**Opioid use history**	
Fentanyl	90 (83%)
Heroin	25 (23%)
Route: smoking	70 (65%)
Route: IV/IM	27 (25%)
History of methadone	81 (65%)
History of buprenorphine	54 (44%)
History of either agent	96 (77%)
**Additional substance use disorders**	
Any non-opioid use disorder	85 (79%)
Stimulant use disorder	67 (62%)
Alcohol use disorder	20 (19%)
Sedative use disorder	13 (12%)
**Additional diagnoses**	
Pregnant/postpartum	16 (15%)
Psychiatric diagnosis	34 (31%)
Chronic pain	31 (29%)
**Hospitalization characteristics (*n* = 124)**	*N* (%) or mean (SD)
**Length of stay**	7.5 days (5.4)
**Primary services**	
Medical	59 (48%)
Surgical	41 (33%)
Medical ICU	5 (4%)
Other	19 (15%)
**Received methadone or buprenorphine**	117 (94%)
Methadone	98 (79%)
Buprenorphine	28 (23%)
**Discharge type**	
Regular	93 (75%)
Patient-directed	31 (25%)

## HOSPITALIZATION CHARACTERISTICS

The mean length of stay was 7.5 days (SD 5.4); medications for opioid use disorder were initiated or continued during 94% of hospitalizations; and 25% of hospitalizations ended in patient-directed discharge (Table 1). Patient-directed discharge occurred most on day 3, before average SAFAO OMEs peaked on day 6. Toxicology, obtained at admission for 67% of hospitalizations (*n* = 84), was most commonly reactive for fentanyl (90%) and methamphetamine (70%).

Average daily COWS peaked on day 1 (mean 8.2, SD 6.4) and decreased daily through the first week of hospitalization, until reaching a minimum on day 6 (mean 4.1, SD 3.2) (Supplement 2). Hospitalizations ending in patient-directed discharge had more severe opioid withdrawal syndrome compared to regular discharge (*p* = 0.04), with 55% of patient-directed discharges exhibiting at least “moderate” withdrawal (COWS > 12) compared to 32% of regular discharges.

## SAFAO REGIMENS

Most hospitalizations had SAFAO initiated on hospital day 1 (62%) or 2 (32%). Four agents were administered: oxycodone (representing 43% of OMEs of SAFAO administered), hydromorphone (39%), fentanyl (16%), and morphine (1%). IV agents were administered during 81% of hospitalizations, representing 51% of total OMEs prescribed. The most frequently selected initial regimen was a combination of oral oxycodone and IV hydromorphone, initiated on day 1 of SAFAO for 88% of hospitalizations.

The average total oral morphine equivalents (OMEs) of SAFAO received per day per patient within the first week of hospitalization was 278 mg (SD 407 mg). OMEs of SAFAO increased daily until peaking on day 6 (409 mg, SD 540 mg) (Supplement 2). Average daily COWS and SAFAO OMEs were inversely correlated during the first week of hospitalization (*r*_s_(5) = − 0.96, *p* = 0.003).

Among hospitalizations for pregnant/post-partum patients, the daily average OME of SAFAO received per patient was 432 mg (SD 520), significantly higher (*t*(60.71) = − 2.38, *p* = 0.02) than non-pregnant/post-partum patients (259 mg, SD 386). The daily average OME administered to surgical patients versus non-surgical patients was statistically equivalent (*t*(461.96) = − 0.45, *p* = 0.65).

## ADVERSE EVENTS AMONG PATIENTS RECEIVING SAFAO

Five hospitalizations (4%) involved oversedation adverse events (Table 2), including four grade 3 (“difficult to arouse”) and one grade 4 (“life-threatening”) adverse events. Two patients who experienced adverse events reported only using heroin. Two events were attributed to in-hospital substance use and one to methadone titration. In the remaining two adverse events, SAFAO use alongside additional sedating medications may have contributed to oversedation. Secondary adverse events (Table 2) were only experienced during the same five hospitalizations with oversedation adverse events. These included two falls, one intubation, and three administrations of naloxone. All patients who experienced adverse events were diagnosed with multiple use disorders.

**Table 2:** Adverse Events (AEs) That Occurred During Hospitalizations in Which SAFAO Was Administered for Opioid Withdrawal Syndrome (*n* = 5 Hospitalizations)

Hospitalization AEs	Sedation grade	Documented reason for sedation	Total naloxone dose received	AEs related to SAFAO?	Opioid use	Comorbid use disorders	Co-administered sedating medications
Sedation	3	Methadone titration	NA	Unlikely	Heroin (intranasal)	Stimulant Tobacco	Gabapentin Methadone
Sedation Fall	3	Treatment of concurrent alcohol withdrawal	NA	Possibly	Fentanyl	Alcohol	Benzodiazepines Gabapentin Hydroxyzine Phenobarbital Methadone
Sedation Fall Naloxone	3	In-hospital substance use	0.1 mg IV	Unlikely	Fentanyl (smoking)	Tobacco	Benzodiazepines Hydroxyzine
Sedation Naloxone Intubation	4	Polypharmacy with sedating medications	1.2 mg IV	Probably	Heroin (IV/IM, smoking)	Stimulant	Benzodiazepines Buprenorphine Gabapentin Haloperidol Ondansetron
Sedation Naloxone	3	In-hospital substance use	0.16 mg IV	Unlikely	Fentanyl (intranasal)	Alcohol Benzodiazepines	Benzodiazepines Diphenhydramine Methadone

## DISCUSSION

The use of SAFAO to treat opioid withdrawal syndrome in the acute care setting represents an expansion beyond current society guidelines, which endorse using methadone, buprenorphine, and non-opioid adjuncts.^24^ In this initiative to improve inpatient management of opioid withdrawal syndrome by an ACT, offering SAFAO to alleviate withdrawal did not dissuade the majority of patients from receiving medications for opioid use disorder during hospitalization. Despite this high rate of using methadone or buprenorphine, adequate treatment of opioid withdrawal syndrome required high doses of SAFAO, including IV agents. This supports the observation that methadone or buprenorphine alone may be insufficient to treat acute opioid withdrawal syndrome.^17,25–27^ The inverse correlation between OMEs of SAFAO and COWS suggests this practice may have alleviated opioid withdrawal. Notably, pregnant and post-partum patients received higher SAFAO doses, which may reflect pregnancy-related augmentation of drug metabolism and/or more aggressive treatment of this sub-population.^28^

Adverse events resulting directly from SAFAO were rare among these 124 hospitalizations. In-hospital substance use contributed to two adverse events and may have reflected under-treated withdrawal.^29^ Patients receiving additional sedating medications and those using only heroin may be at increased risk for adverse events. This latter finding suggests that despite high levels of fentanyl contamination in the heroin supply, patients with heroin use disorder may have lower opioid tolerance than those intentionally using fentanyl.^30^

Patient-directed discharge was common in our cohort despite SAFAO use, although these discharges mostly preceded administration of peak doses. Patient-directed discharge may have been driven in part by opioid withdrawal, suggested by elevated COWS relative to patients with regular discharge and as shown in prior studies. ^3,5,31^ The early timing of patient-directed discharge in this cohort suggests a need for more proactive management of opioid withdrawal syndrome early in hospitalization.

One limitation of this evaluation is that we are unable to distinguish use of SAFAO for opioid withdrawal syndrome versus pain. The finding that surgical and non-surgical patients received statistically equivalent OMEs of SAFAO is reassuring that acute surgical pain does not likely account for a substantial portion of SAFAO administered in this cohort. Additionally, we have no comparison group of patients with opioid withdrawal syndrome who did not receive SAFAO, precluding inference of causality between SAFAO administration and relevant outcomes. We excluded patients with hospitalization < 24 h, reasoning that these patients did not stay long enough to be evaluated and treated by the ACT. This may have falsely lowered our rate of patient-directed discharge. Lastly, our observations may not be fully generalizable to institutions without an ACT.

Clinicians seeking to adopt this practice should note the wide range of OMEs required by patients in this study. Many patients require aggressive titration of SAFAO, including significant doses of IV opioids, while others are comfortable with more modest dosing. This variance is likely referable to several factors, including variable baseline opioid tolerance, acute and chronic disease states, and fluctuations in the potency of the illicit opioids supply. We recommend frequent reassessment and titration based on patients’ subjective symptoms (including cravings), COWS scores, and physical examination. Pregnant and post-partum patients may require particularly high doses. Though clinicians should exercise caution in treating patients with concurrent withdrawal syndromes and those who report using only heroin, our study demonstrates that this practice may be generally well-tolerated in appropriately selected patients. We recommend that hospitals seeking to implement a similar practice educate front-line clinical staff regarding rationale, patient selection, and safety considerations.

Further work should seek to optimize opioid withdrawal syndrome treatment early in hospitalization, develop protocolized approaches for the safe use of SAFAO to treat opioid withdrawal syndrome, and assess the impact of SAFAO-based treatment on linkage to medications for opioid use disorder post-discharge. A standardized EHR order set and accompanying protocol are currently in development at our hospital; future work will evaluate their effectiveness for alleviating opioid withdrawal syndrome.

## Supplementary Information

Below is the link to the electronic supplementary material.

Supplementary file1 (DOCX 248 KB)

## Data Availability

The data supporting the findings of this study are considered protected health information under HIPAA regulations and cannot be publicly shared. Researchers interested in accessing a de-identified version of the data for further analysis must submit a data use agreement and obtain approval from the Institutional Review Board. Contact the corresponding author for further inquiries regarding data access procedures.
