# Supplementary material for: Treatment of Inpatient Opioid Withdrawal with Short-Acting Full Agonist Opioids at a Safety-Net Hospital
Source: J Gen Intern Med. 2025 Jan 6;40(12):2890–5. doi: 10.1007/s11606-024-09321-5 (PMC12463741; doi:10.1007/s11606-024-09321-5)
Supplement: Supplementary file 1 — Supplementary file1 (DOCX 248 KB) [file 11606_2024_9321_MOESM1_ESM.docx]

**Supplementary Information**

**Supplement 1: ACT Guidance: Use of Short-Acting Opioids to Treat Opioid Withdrawal**

Introduction and rationale:

Patients who use heroin, fentanyl, and other potent synthetic opioids on a chronic daily basis often have high levels of physiologic opioid tolerance. When these patients are admitted to the hospital, they frequently require short-acting full agonist opioids to prevent and treat opioid withdrawal. This can be done in conjunction with methadone titrations, low-dose buprenorphine starts, or as standalone treatment during hospitalization for patients who defer methadone or buprenorphine for opioid use disorder. Preventing and treating withdrawal is necessary to enable treatment of acute medical issues, reduce patient-directed discharges and in-hospital substance use, and relieving suffering from pain and withdrawal. Additionally, maintaining opioid tolerance in the hospital is important to lower risk of overdose for patients who return to use.

For patients in acute heroin/fentanyl withdrawal:

- Short-acting opioids including IV opioids are appropriate and should be titrated aggressively to control withdrawal symptoms.
- Some patients may require very large doses of opioids to control their withdrawal symptoms, but tolerance can vary significantly between patients. Oxycodone 20mg PO q3h PRN and hydromorphone 2mg IV q2h PRN are reasonable starting doses for most patients. Start lower if your patient is at increased risk of over-sedation (e.g. concurrently receiving other sedating medications, acute neurological illness, etc.). Encourage teams to titrate up if the patient is uncomfortable and is not experiencing respiratory depression.
- This may not apply to patients with pill-based opioid use disorder, who use opioids intermittently, and/or in relatively small amounts.

For patients started on medications for opioid use disorder:

- Once a therapeutic dose of buprenorphine is achieved, full agonists can be stopped.
- For patients starting on methadone, once withdrawal is stabilized, begin tapering short-acting opioids – especially IV agents – with each increase in methadone dose. This requires early and proactive expectation-setting with the patient.
- If there is acute pain in addition to opioid use disorder/opioid withdrawal syndrome, support teams in making a plan to use short-acting agents to treat this problem (e.g. as-needed doses prior to wound care) along with a plan to taper as appropriate.

For patients planning to return to use:

- Continue short-acting opioids throughout the hospital stay to prevent withdrawal and maintain tolerance. Set expectations with patients that these will be stopped on discharge.
- In general, try to reduce IV opioid use in favor of PO once withdrawal is controlled. However, some patients may require ongoing IV opioid agonist treatment to remain in the hospital for treatment of their acute medical issues. In these situations, please document clearly your rationale for continuing IV agents and consider discussing with the primary team.


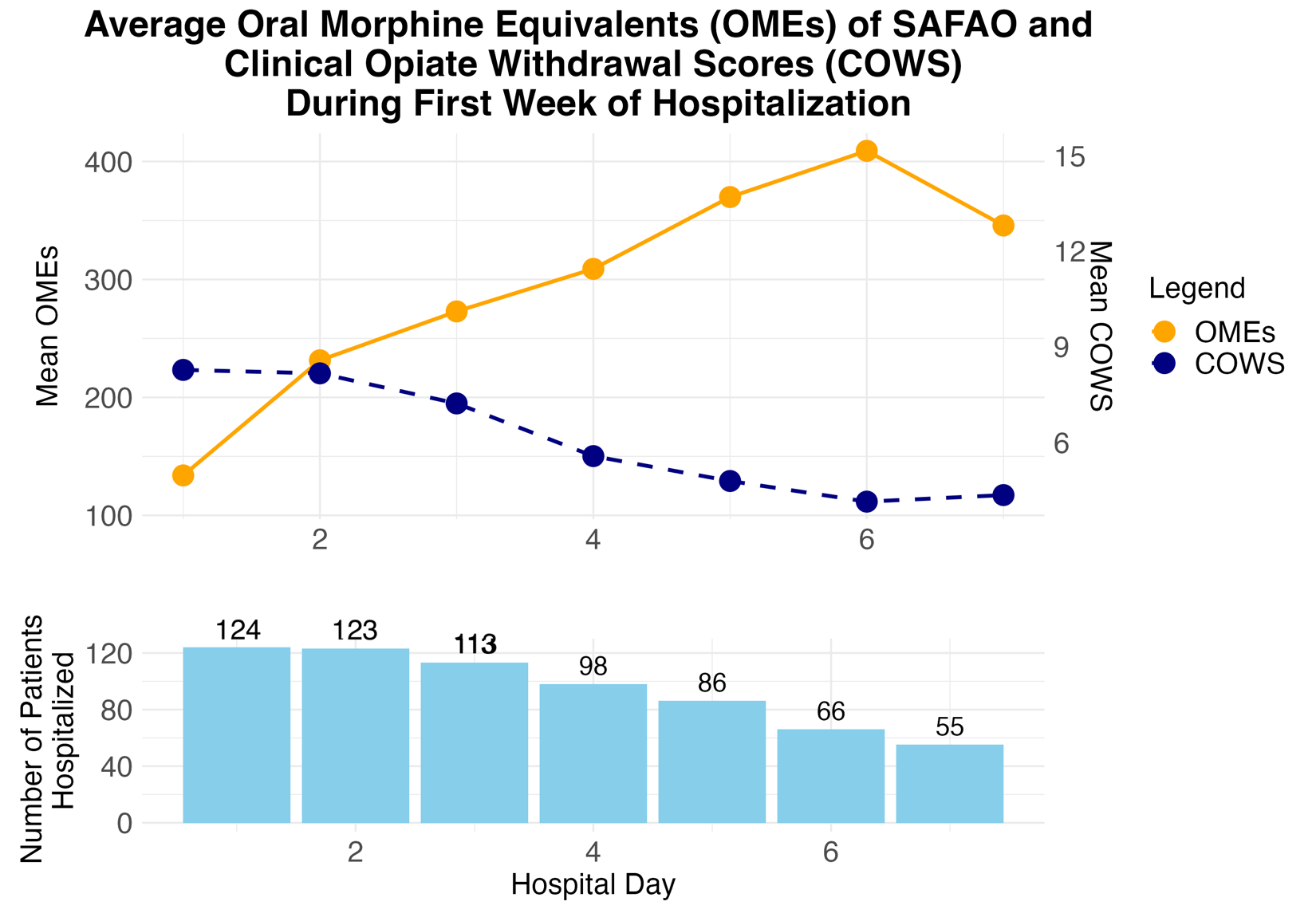
**Supplement 2**
